# Wearable Temperature Sensor Enhanced Volatilomics Technique for Swift and Convenient Detection of Latrogenic Botulism

**DOI:** 10.1002/advs.202411738

**Published:** 2024-12-16

**Authors:** Xiaoyang Li, Yufei Yan, Chenyi Hu, Jing Wang, Jinlin Wang, Hao Yang, Daxiang Cui, Wenwen Xin, Shan Gao, Han Jin

**Affiliations:** ^1^ State Key Laboratory of Pathogen and Biosecurity Academy of Military Medical Sciences Beijing 100071 China; ^2^ Institute of Micro‐Nano Science and Technology & National Key Laboratory of Advanced Micro and Nano Manufacture Technology School of Electronic Information and Electrical Engineering Shanghai Jiao Tong University Shanghai 200240 P. R. China; ^3^ Medical School Henan University Kaifeng Henan Province 475004 P. R. China; ^4^ National Engineering Research Center for Nanotechnology Shanghai 200241 P. R. China; ^5^ Wuzhen Laboratory Tongxiang Zhejiang Province 314500 P. R. China

**Keywords:** Botulinum neurotoxin (BoNT) poisoning, GelMA film, Volatile markers, Wearable temperature sensor, YSZ‐based gas sensors

## Abstract

Accurately assessing potential side effects following botulinum neurotoxin (BoNT) injection remains a formidable challenge. To address this issue, an innovative approach is developed that combines a wearable temperature sensor with a sophisticated volatilomics technique, aimed at facilitating the rapid and convenient prediction of potential physical discomfort related to latrogenic botulism. The investigation identifies five volatile organic compounds (VOCs)—acetone, styrene, ethanol, 2‐pentanone, and n‐butano—as promising markers indicative of BoNT poisoning. Specifically, a handheld breath analyzer, featuring a yttrium stabilized zirconia (YSZ)‐based gas sensor array, alongside a wearable temperature sensor integrated with a bio‐compatible methacrylated gelatin (GelMA) sensing film, are developed to simultaneously monitor breath signal variations and body temperature fluctuations. Preliminary animal testing validates the effectiveness of the integrated approach, achieving an accuracy exceeding 91.2% in early detection of physical discomfort associated with BoNT poisoning. These promising findings represent a significant advancement towards the early identification of BoNT‐related issues, enabling timely intervention and improved management strategies.

## Graphical abstract

A wearable temperature sensor enhanced volatilomics technique is developed to facilitate the rapid and convenient prediction of potential physical discomfort following botulinum neurotoxin (BoNT) injection. Through simultaneously monitoring the variation of body temperature and breath signals, this innovative strategy achieves an accuracy exceeding 91.2% in the early detection of physical discomfort associated with BoNT poisoning.

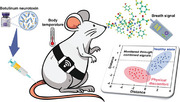

## 1 Introduction

Botulinum neurotoxin (BoNT) is a group of neurotoxins produced by the bacterium Clostridium botulinum and is widely used for cosmetic applications.^[^ ^1^ ^]^ BoNT restricts neuronal communication by inhibiting the release of acetylcholine at the motor end‐plate and thus, suppressing muscle contraction.^[^ ^2^ ^]^ Beyond the well‐known use in cosmetic procedures (like Botox for reducing wrinkles), BoNT are also used in medical treatments to manage various conditions,^[^ ^3^ ^]^ such as muscle spasm,^[^ ^4^ ^]^ overactive bladder,^[^ ^5^ ^]^ strabismus,^[^ ^6^ ^]^ etc. Although the injection of BoNT to induce muscle inactivation is recognized as a safe and effective procedure, it still also may lead to impaired or complete loss of respiratory function, namely Iatrogenic botulism, one of the most common types of BoNT poisoning.^[^ ^7^ ^]^ Symptoms of Iatrogenic botulism typically include blurred vision, difficulty swallowing, muscle weakness, and breathing difficulties.^[^ ^8^ ^]^ If these symptoms occur, the person should seek medical attention immediately and be administered botulinum antitoxin as soon as possible to neutralize the toxin for preventing further damage.^[^ ^9^ ^]^ Considering the side effects, it is crucial to discriminate any potential discomfort following a Botox injection.

To date, mouse bioassay remains the “gold standard” for detecting potential side effects which involves injecting a specified amount of BoNTs into mice.^[^ ^10^ ^]^ Despite its notable reliability and sensitivity, this method has significant drawbacks that limit its practical application for rapid detection.^[^ ^11^ ^]^ These include the necessity for mice, costly animal facilities, highly skilled personnel, and extended measurement times, often spanning several days.^[^ ^12^ ^]^ Over the past decades, substantial focus has been placed on developing alternative methods to animal testing.^[^ ^13^ ^]^ These advancements include a range of innovative techniques such as enzyme‐linked immunosorbent assays (ELISA),^[^ ^14^ ^]^ nucleic acid‐based approaches,^[^ ^15^ ^]^ electrochemiluminescence methods,^[^ ^16^ ^]^ lateral flow immunoassays,^[^ ^17^ ^]^ surface‐enhanced Raman scattering (SERS),^[^ ^18^ ^]^ and surface acoustic wave (SAW) immunosensors,^[^ ^19^ ^]^ among others. Compared to traditional mouse bioassay, these alternative sensing techniques provide the benefit of relatively rapid BoNT analysis, although they may require more complex sample pre‐processing.^[^ ^20^ ^]^ Nevertheless, while the aforementioned approaches effectively detect the presence of BoNT, they are limited in their ability to assess or predict potential side effects that may occur following BoNT injection. This is due to the fact that some individuals may experience discomfort even at low doses, while many others report discomfort at higher doses.^[^ ^21^ ^]^

Breathing difficulties are commonly reported as a major symptom of BoNT poisoning. This observation inspires that monitoring changes in specific metabolized volatile organic compounds (VOCs) could indicate excessive BoNT injection, as BoNT poisoning impacts breath metabolism and its related metabolites. Based on the principle of volatilomics, heightened levels of these volatile organic compounds (VOCs) could provide a rapid and convenient way to evaluate any physical discomfort after receiving a BoNT injection.^[^ ^22^ ^]^ On the other hand, considering that BoNT poisoning can impact the body's blood circulation, it is plausible that the toxin may induce vasoconstriction and impaired blood flow, leading to inadequate blood supply to the extremities and a resultant sensation of coldness.^[^ ^23^ ^]^ In other words, fluctuations in body temperature are expected during BoNT poisoning. Building on these expectations, we aim to develop a rapid and convenient method for predicting potential BoNT poisoning in cosmetic applications by simultaneously monitoring changes in specific VOCs of exhaled breath and body temperature variations. To achieve this goal, we will specifically screen VOCs that could serve as indicators for BoNT poisoning. Simultaneously, we will develop a handheld device and a wearable flexible sensor to monitor changes in specific VOCs and body temperature, respectively. Ultimately, a thorough animal test to assess the effectiveness of predicting BoNT poisoning after toxin injection will be particularly implemented in this research.

## 2 Results and Discussion

### 2.1 General Strategy of the Research

**Figure 1:**
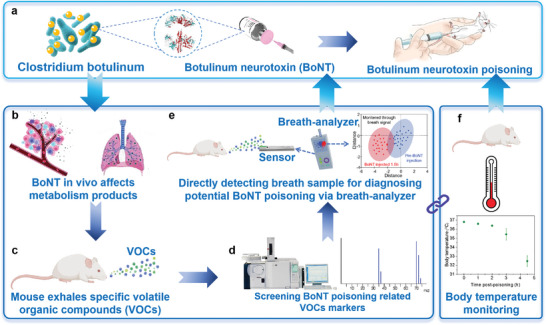
Illustration of overall research strategy. a) Administrating mice with botulinum neurotoxin (BoNT) at a controlled level to induce a model of botulism; b) BoNT influences metabolic processes in the body, resulting in c) the elevation of specific volatile organic compounds (VOCs) in exhaled breath; d) confirming the chemical structure of VOCs markers through GC‐MS; e) assessing exhaled breath alongside f) monitoring body temperature fluctuations to non‐invasively evaluate any physical discomfort following a BoNT injection.

To investigate botulinum neurotoxin (BoNT) effects, a controlled concentration derived from Clostridium botulinum was administered to mice for inducing a model of botulism (**Figure** **1**a). Since BoNT poisoning can lead to respiratory difficulties, impacting breath metabolism significantly, it is believed that certain VOC levels in exhaled breath may increase as a result (**Figure** 1b,c). These VOCs were comprehensively analyzed using gas chromatography‐mass spectrometry (GC‐MS) techniques (Figure 1d). To non‐invasively and promptly assess any physical discomfort following a BoNT injection, breath samples from mice containing these VOC markers were meticulously analyzed using a handheld device. Additionally, the body temperature of the mice was monitored using a flexible temperature sensor to further enhance detection accuracy (Figure 1e,f).

### 2.2 Screening Potential VOC‐Based Indicators in Exhaled Breath for Detecting BoNT Poisoning

**Figure 2:**
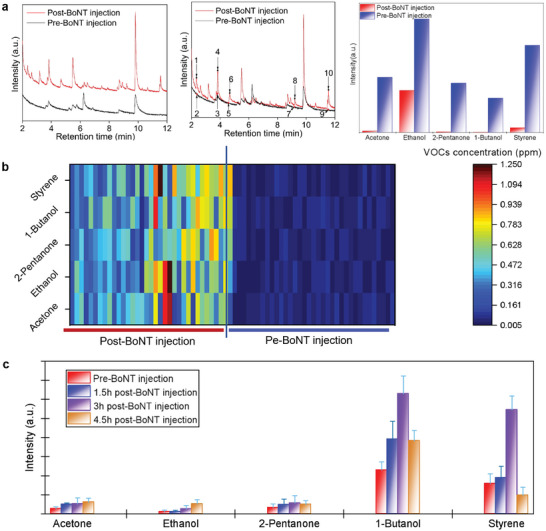
Screening VOC indicators for the early detection of BoNT poisoning. a) Representative chromatograms of mice following BoNT injection and healthy mice, demonstrating the increased intensity of VOC biomarkers in the breath samples of BoNT‐poisoned subjects compared with healthy controls. 1,2: acetone; 3,4: ethanol; 5,6: 2‐pentanone; 7,8:1‐butanol; 9,10: styrene; b) heatmap depicting the intensity variations between BoNT‐poisoned and control subjects. The squares correspond to VOC markers in breath samples, with color gradients indicating their abundance (peak area); c) temporal variations in breath biomarker levels after BoNT injection.

To assess the feasibility of using specific VOCs for early warning of side effects following BoNT injection, we initially screened potential VOC‐based indicators in exhaled breath for detecting BoNT poisoning. In this study, mouse was selected as the research model. Breath components derived from various mice were captured using sorbent material and analyzed with GC‐MS system. **Figure** **2**a displays representative chromatograms for healthy mice and mice following BoNT injection, highlighting numerous peaks that correspond to different VOCs in breath samples. 5 peaks with significant differences (*p* < 0.001) between BoNT injected and control groups were identified as volatile markers, namely, acetone; ethanol; 2‐pentanone, 1‐butanol and styrene. As can be confirmed that the BoNT‐administrated group exhibited higher VOCs concentrations compared to controls. Then, the differences in concentrations of these 5 VOC markers between the BoNT‐injected subjects (post‐BoNT injection) and the control subjects (pre‐BoNT injection) were further systematically studied and visually displayed in the form of heatmap, as shown in Figure 2b. The elevated intensity of VOC markers in breath samples from the BoNT‐injected group indicates a shift in metabolic profiles following BoNT administration. Figure 2c depicts the variation of the level for these 5 volatile markers on the poisoned time. An increase in the 5 volatile markers was observed over time, with a particularly notable rise three hours later following the BoNT injection. However, by the fourth hour, a decrease in some VOCs was noted, likely due to respiratory difficulties in the mice resulting from severe BoNT poisoning. The respiratory difficulties observed in mice injected with BoNT for 4 h hinder their ability to exhale adequate volatile markers, resulting in reduced levels of breath markers. Finally, specificity of using these 5 VOC markers in identifying BoNT poisoning was verified. Figure S1 and Tables S1 and S2 (Supporting Information) illustrate that the group of VOC markers was absent in breath samples from mice afflicted with ricin or tetrodotoxin poisoning, as well as those experiencing hypoxia. This finding suggests a remarkable specificity for BoNT detection using volatilomics technique. In conclusion, these results compellingly demonstrate that significant distinctions between BoNT‐poisoned and healthy groups can be effectively classified using these VOC markers, highlighting the promising potential for a non‐invasive method to assess physical discomfort following a BoNT injection.

### 2.3 Tracking Volatile Markers Through Electrochemical Gas Sensor Array

**Figure 3:**
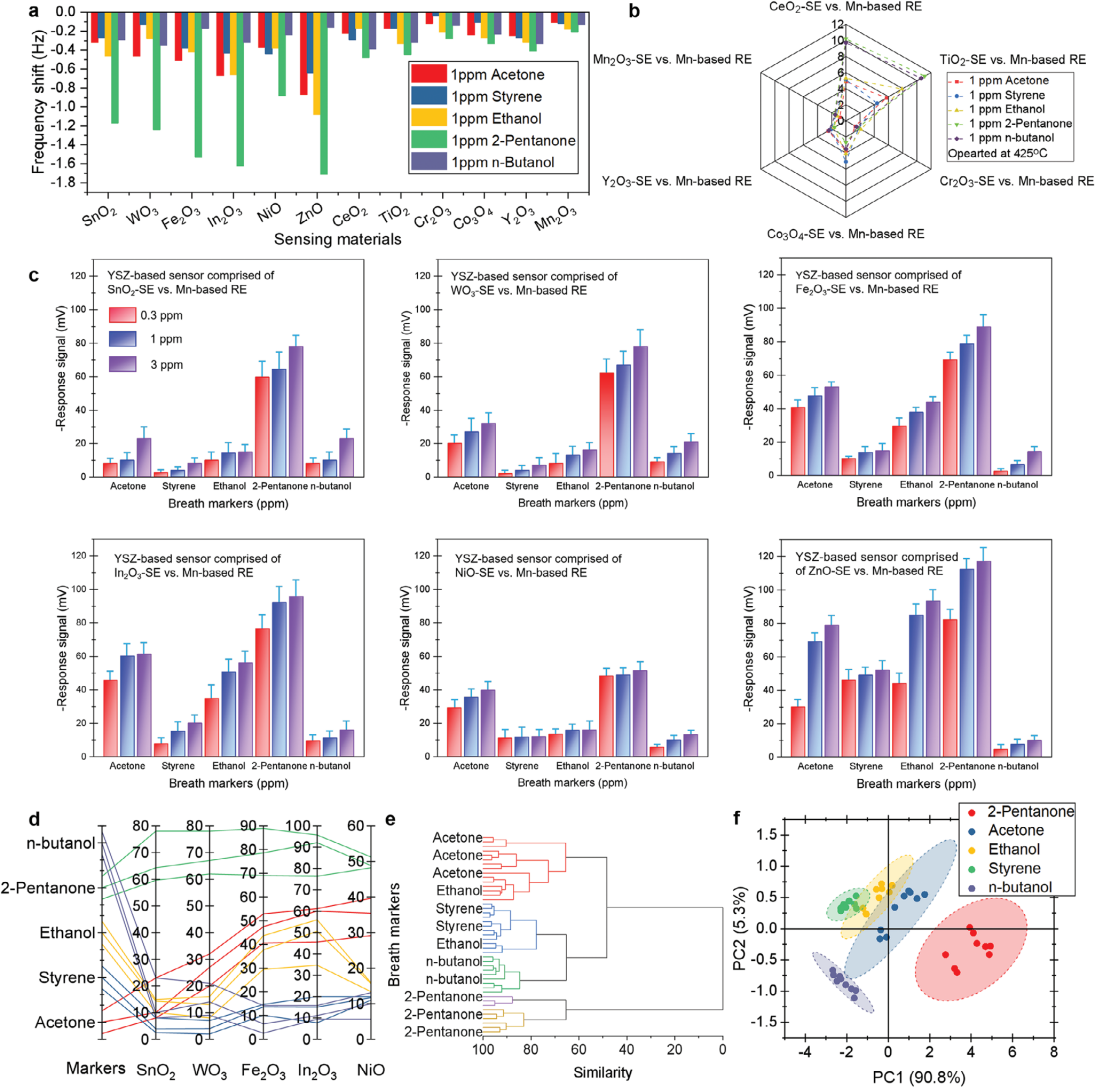
Sensing characteristics towards each volatile marker. a) Comparison of the gas adsorption capacity for various metallic oxide‐based sensing materials towards acetone, styrene, ethanol, 2‐pentanone, and n‐butanol; b) response patterns and c) cross‐sensitivity of the sensors using SnO_2_, WO_3_, Fe_2_O_3_, In_2_O_3_, NiO, and ZnO‐SEs (versus Mn‐based RE) toward the targeted volatile markers at different concentration; d) parallel coordinate plots of 5 response patterns extracted from all VOC gas response measurements for acetone, styrene, ethanol, 2‐pentanone, and n‐butanol, corresponding to the purple, green, yellow, blue, and red group lines, respectively; Differentiate efficiency of the sensor array toward five kinds of VOC‐based indicators that presented in the form of e) hierarchical clustering dendrogram and f) PCA map.

For the purpose of efficiently tracking the variation of aforementioned volatile markers (i.e., acetone, styrene, ethanol, 2‐pentanone, n‐butanol), elecrochemical gas sensors comprised of diverse sensing materials were developed and characterized. We started by comparing the gas adsorption capabilities of different metallic oxides to identify the sensing materials that exhibit the greatest affinity for volatile marker adsorption. This approach allows for the precise detection of VOC‐based indicators with exceptional sensitivity. As shown in **Figure** **3**a, among all the examined metallic oxides, SnO_2_, WO_3_, Fe_2_O_3_, In_2_O_3_, NiO, and ZnO demonstrated relatively larger frequency shift when exposed to the studied volatile markers. These significant shifts indicate a high affinity for specific gas species, suggesting that these VOC markers preferentially adsorb on the surfaces of SnO_2_, WO_3_, Fe_2_O_3_, In_2_O_3_, NiO, and ZnO. In simple terms, the aforementioned metallic oxide are highly promising and effective candidates for high‐performance tracking the VOC markers. Then, several yttrium stabilized zirconia (YSZ)‐based electrochemical gas sensors were created by using the selected metallic oxides as sensing electrodes (SEs) and a Mn‐based reference electrode (RE), capitalizing on the impressive sensing performance of these sensors in challenging conditions, such as high humidity. These sensors' performance in detecting VOC markers was rigorously evaluated to confirm our hypothesis. Additionally, operating parameters, including fabrication and operating temperatures, were optimized. According to the results illustrated in Figures S2 and S3 (Supporting Information), optimal response behavior was observed at the fabrication and operational temperatures of 1050 and 425 °C, respectively. Figure 3b,c and Figure S4 (Supporting Information) illustrate the sensing characteristics that are measured at the optimal operating parameters. In brevity, an apparent response signal was confirmed for the sensors comprised of SnO_2_, WO_3_, Fe_2_O_3_, In_2_O_3_, NiO, ZnO‐SEs, and Mn‐based RE (Figure 3b). In contrast, the sensors using other metallic oxides (e.g., CeO_2_, TiO_2_, Cr_2_O_3_, Co_3_O_4_,Y_2_O_3_, Mn_2_O_3_) SEs displayed minor response signal When exposed to the targeted VOC markers (Figure S4, Supporting Information). This finding aligns with the result shown in Figure 3a and directly supports our hypothesis. The 90% response and recovery times for these sensors are ≈16 and 25 s, respectively (Figure S5, Supporting Information). This rapid response is advantageous for detecting rapid fluctuations in VOC‐based indicators. Particularly, due to the fact that water vapor tends to desorb at operating temperatures exceeding 350 °C, combined with the minimal impact of water vapor on the ionic conductivity of the YSZ‐based electrolyte, these sensors demonstrated excellent response stability and resistance to breath moisture during the monitoring of volatile markers (Figures S6 and S7, Supporting Information). These impressive sensing characteristics indicate the feasibility of tracking trace amount of VOC‐based indicators in practical conditions. Furthermore, the distinct response behavior of these sensors highlights their capability to provide multidimensional response patterns in sensing applications (Figure 3c). The multidimensional response patterns will facilitate the effectiveness of pattern recognition algorithms, thereby significantly improving gas classification capabilities.

An electrochemical sensor array, featuring optimal metallic oxides‐SEs and a Mn‐RE, was finally designed to efficiently differentiate the aforementioned volatile markers. Distinct characteristics corresponding to five different gas species were accurately extracted using fitting parameters that reflect their respective response curves. As illustrated in Figure 3d, each line connects parameter values along their respective axes—representing the sensing responses from each sensor—forming a distinct point within the 5‐dimensional parameter space. This visualization provides valuable insights into the clustering patterns of gas responses at specific parameters, revealing the convergence of measurements. The differentiated efficiency of the sensor array towards targeted VOC markers was depicted in the form of hierarchical clustering dendrogram and PCA map. It is reasonable to deduce that the developed sensor array demonstrated satisfactory recognition capability, as evidenced by the clear clustering of targeted volatile markers (Figure 3e) and minimal overlap between each gas species group (Figure 3f). Meanwhile, the power consumption of the sensor is ≈24.6 mW, which is an acceptable level for a handheld breath analyzer. Conclusively, the developed sensor array exhibited great promise for high‐performance tracking the variation of VOC‐based indicators.

### 2.4 GelMA‐based Bio‐Compatible Flexible Temperature Sensing Film for Accurate Recording Body Temperature

**Figure 4:**
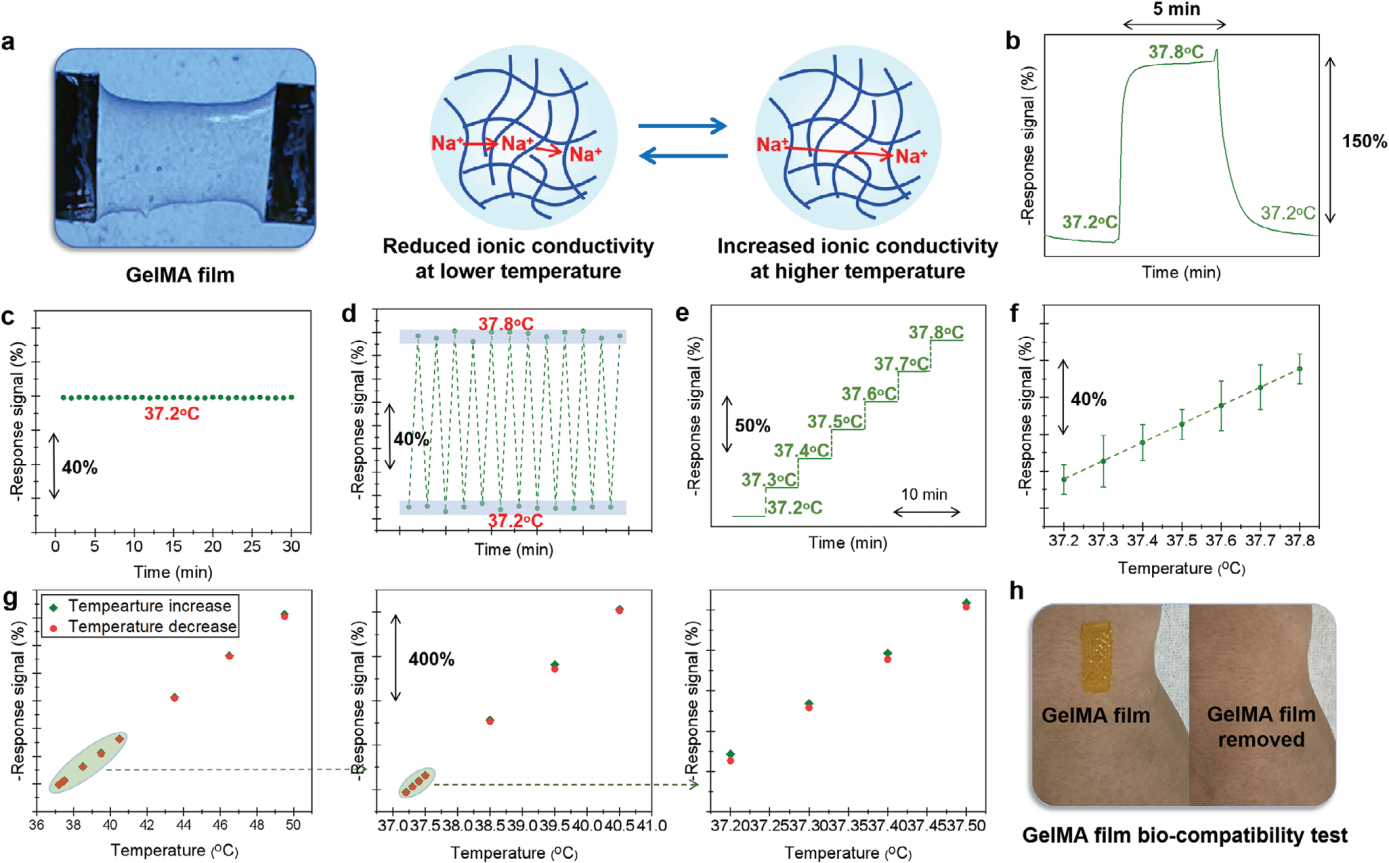
Characterizing temperature sensing properties of GelMA film. a) Photographic image and temperature sensing principle of GelMA film; b) Response transient of GelMA film to thermal shock induced by electronic thermostat; c) Stability test within 30 days at the temperature of 37.2 °C; d) Response repeatability within the temperature range of 37.2 °C and 37.8 °C; e) Step response and f) dependence of response signal on the temperature in the range of 37.2 to 37.8 °C; g) Response variation measured at the temperature increase and decrease, in the range of 37.2 to 49.5 °C; h) Bio‐compatibility verification of GelMA sensing film.

In light of the fact that body temperature fluctuations typically vary by ≈0.1 °C, and to achieve a comfortable measurement experience, high sensitivity and desirable deformability as well as good bio‐compatibility are particularly required for the sensing material. In response to the demand, we selected methacrylated gelatin (GelMA) as the candidate for tracking body temperature variation. GelMA is widely recognized for its bio‐friendly,^[^ ^24^ ^]^ flexibility,^[^ ^25^ ^]^ and temperature sensitive,^[^ ^26^ ^]^ making it an excellent choice for this application. Herein, a sensing film made by GelMA was obtained through spin coating and its sensing characteristics was thoroughly studied (**Figure** **4**a). The GelMA film demonstrated quick 90% response/recovery rate (around 11s and 27s, respectively) and desirable response stability as well as acceptable repeatability (Figure 4b‐d). Moreover, acceptable temperature sensitivity (2.47 °C^−1^) as well as the linear relationship between temperature and response signal were further confirmed (Figure 4e,f). It should be particularly noted that the fabricated GelMA film exhibited approximately zero hysteresis regardless the measured temperature range (Figure 4g,h) and Figure S8 (Supporting Information) compared the bi‐compatibility of the GelMA film with that of the commonly used ecoflex film by observing the physical status of the skin covered with GelMA film or ecoflex film for 2h. After attaching the ecoflex film, the skin showed signs of redness, while the area covered with the GelMA film remained unaffected. This indicates that the GelMA film demonstrated outstanding bio‐compatibility. It is important to highlight that GelMA generally exhibits greater sensitivity to temperature than to pressure. Furthermore, since the GelMA sensing film is spin‐coated onto a glass substrate, any interference from mouse movement during temperature sensing is minimal. Based on these results, it is inferred that the GelMA film is effective for monitoring body temperature variations potentially associated with BoNT poisoning.

### 2.5 Real‐Time Monitoring Abnormal Physiological Parameters After BoNT Injection Using a Handheld Breath‐Analyzer and a Wearable Temperature Sensor

Building on the foundation of high‐performance YSZ‐based VOCs sensor array and bio‐compatible GelMA‐based temperature sensing film, we developed a handheld breath analyzer and a wearable temperature sensor to monitor changes in physiological parameters. Figures S9–S11 (Supporting Information) demonstrate the photographic image, detection accuracy, and circuit design of the breath analyzer or wearable temperature sensor. As presented in Figure S9a (Supporting Information), the breath‐analyzer is mainly composed of the following parts: sensor module (YSZ‐based VOCs sensor array), power module and data processing module (main cipher unit, MCU). Meanwhile, the proposed wearable temperature sensor that integrated GelMA film and flexible circuit (containing power battery, Bluetooth module, MCU, Figure S9b, Supporting Information) was directly placed on the naked skin of the mouse for real‐time monitoring of the body temperature fluctuation. Figure S9c,d (Supporting Information) compares the data deviation of the recorded value through a breath‐analyzer (vs GCMS) or wearable temperature sensor (vs electronic thermometer). The identical results implied that both the breath‐analyzer and wearable temperature sensor demonstrated high accuracy in recording data.

**Figure 5:**
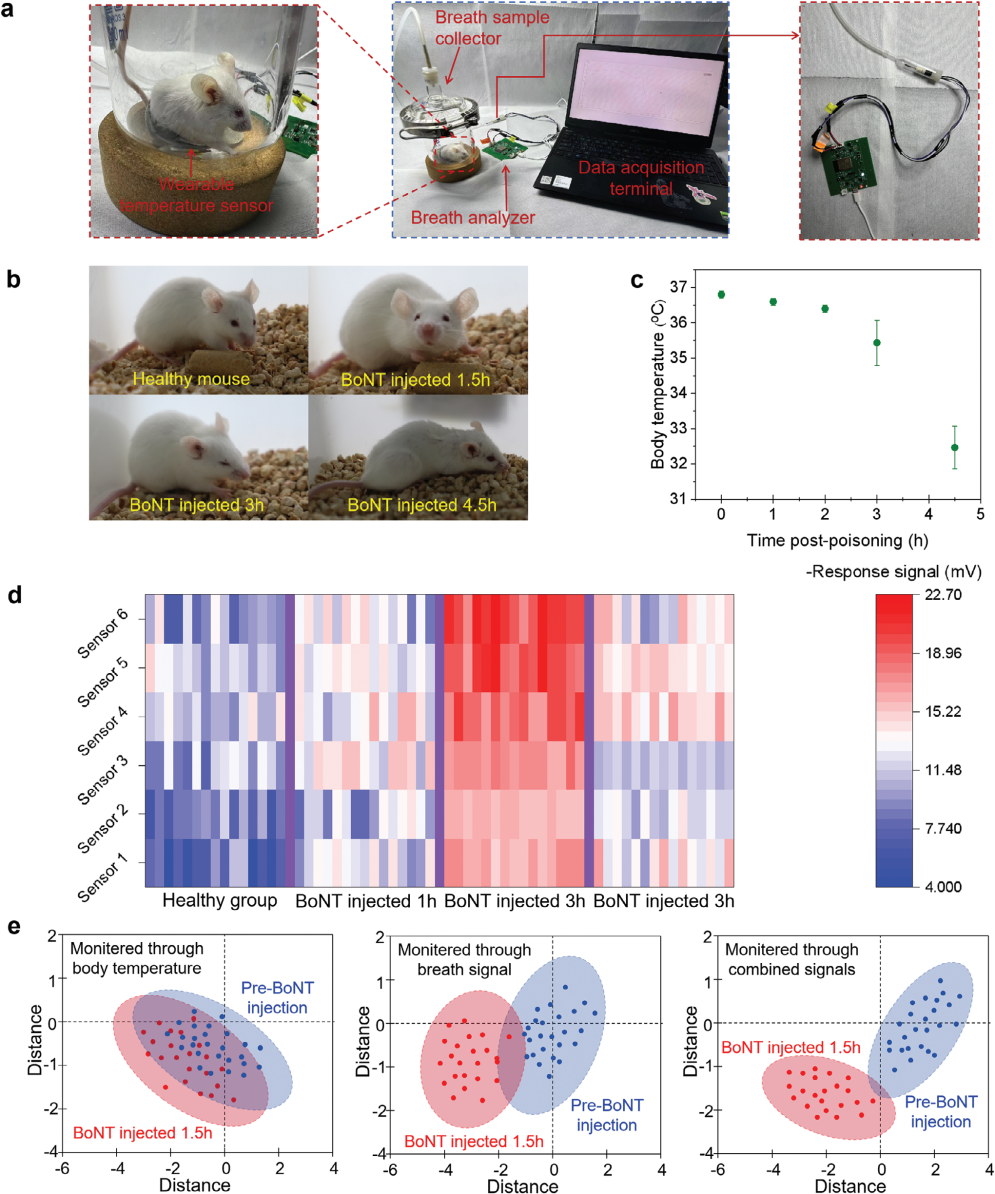
a) Illustration for simultaneously recording breath signal and body temperature fluctuation of mice; b) photographic images of mice pre‐ or post‐BoNT injection; Variation of c) body temperature and d) breath signal on the time post‐BoNT injection; e) PCA map obtained from body temperature signal, breath signal, and combined signal.

**Figure** **5**a illustrates the details of collecting external physical signs and breath signal as well as body temperature of the mice both before and after BoNT injection. Notably, significant physical symptoms, including limb weakness and difficulty breathing, were observed in the mouse 3 hours post‐BoNT injection (Figure 5b). Nevertheless, these symptoms were scarcely present in the mouse that was administered BoNT within the first 2 h. This means that in the early stages of BoNT poisoning, evaluating discomfort symptoms based solely on external signs can be challenging. Figure 5c,d presents the variations in body temperature and fluctuations in breath signal, respectively. It is observed that as the duration of poisoning extends, the mice's body temperature gradually decreases, while their breath signal steadily increases. This trend became particularly striking after more than 2 h of poisoning. Given the marked contrast between the breath signals and body temperatures of healthy mice and those administered with BoNT, we anticipated that BoNT poisoning in mice can be effectively identified through breath analysis and will be further enhanced by the associated changes in body temperature. Consequently, by extracting body temperature data from the wearable temperature sensor and breath signals from the breath analyzer as matrix eigenvalue, we correlated and visualized these values in PCA maps. These representations effectively highlighted the variations between different time points following BoNT injection and the healthy state. As depicted in Figure 5e, the recognition accuracy of the PCA pattern utilizing both body temperature and breath signals significantly surpasses that of the PCA maps relying solely on either body temperature data or breath signals. This combined method achieved a superior recognition performance in assessing potential physical discomfort, with an accuracy of exceeding 91.2% (see Figure 5e and Figure S12, Supporting Information). Notably, the combined approach can provide an early warning of potential physical discomfort within 1.5 h after a BoNT injection (Figure 5e and Figure S12, Supporting Information), allowing for timely intervention and better management across various scenarios involving botulism patients.

## 3 Conclusion

To facilitate the rapid and convenient prediction of potential physical discomfort following BoNT injection, a wearable temperature sensor‐enhanced volatilomics technique was developed specifically for cosmetic applications. Through a comprehensive analysis of the impact of BoNT injection on the exhaled breath of mice, five kinds of VOCs were identified as promising indicators for predicting BoNT poisoning. Additionally, we proposed a handheld breath analyzer featuring a high‐performance YSZ‐based gas sensor array and a wearable temperature sensor utilizing a biocompatible GelMA sensing film for real‐time monitoring of breath signal variations and body temperature changes. Preliminary animal test verified the practicability of our method in early warning of potential physical discomfort from BoNT poisoning, achieving an accuracy exceeding 91.2%. Particularity, the integration of a handheld breath‐analyzer and a wearable temperature sensor enables individuals a comfortable and non‐invasive approach to monitor their physical state following BoNT injection, thereby providing the opportunity of timely treatment if needed. In sum, we demonstrated the effectiveness of a combined body temperature and volatilomics approach for rapidly and non‐invasively assessing potential BoNT poisoning, even at a stage with minimal external signs. While further in‐depth research, such as clinical trials, is necessary, these promising results represent a significant step toward the early detection of BoNT‐related issues.

## 4 Experimental Section

The animal experimental protocol was approved by the Animal Ethics Committee (AEC) of Shanghai Jiao Tong University (SJTU), Shanghai, P. R. China (AEC Reference No.: 202001054). Besides, Mr. Xiaoyang Li has given consent for the biocompatibility testing of the hydrogel.

### Screening Volatile Markers for Indicating BoNT Poisoning

To investigate whether botulinum toxin exposure induces changes in the composition and concentration of exhaled breath in mice, the Solid‐Phase Microextraction (SPME) method was employed to collect exhaled breath samples before and after exposure, followed by analysis using Gas Chromatography‐Mass Spectrometry (GC‐MS). To account for the influence of different exposure routes on the results, mice subjected to intraperitoneal injection were tested. The procedure is detailed as follows: Twelve male BABL/c mice were randomly divided into two groups of six, designated as the injection group and the gavage group. Initially, exhaled breath samples were collected and analyzed for composition and concentration prior to exposure. For the injection group, 0.5 mL of botulinum toxin was administered intraperitoneally in a sequential manner, and the mice were subsequently placed in a collection chamber for gas sampling, with supplemental oxygen provided every 30 minutes at a flow rate of 15 mL. For the gavage group, the toxin was diluted 1:1 with milk, and 0.5 mL was administered every 0.5 hours. After 1 h, the mice were placed in a collection chamber for gas sampling, with supplemental oxygen provided every 30 minutes at 15 mL. Post‐exposure, exhaled breath samples were collected and analyzed. Paired t‐tests were employed to assess changes in the composition of exhaled breath before and after exposure, with significant changes in components identified as potential biomarkers for botulinum poisoning.

### Verifying the Specificity of Selected Volatile Markers

To validate the specificity of the identified breath biomarkers for botulinum poisoning, similar analyses were performed on exhaled breath from mice exposed to hypoxia, ricin, and tetrodotoxin. Eighteen male BABL/c mice were randomly assigned to three groups of six: ricin poisoning, tetrodotoxin poisoning, and hypoxia. Each mouse within the groups was numbered and analyzed in sequence. The procedure involved: For the ricin and tetrodotoxin groups, 0.5 mL of toxin diluted in PBS was administered intraperitoneally in a sequential manner, followed by placement in a collection chamber for gas sampling, with oxygen supplied every 30 min. For the hypoxia group, mice were exposed to a sealed chamber for 3 h without additional oxygen, then placed in a collection chamber in sequence. All exhaled breath samples were collected using SPME and analyzed by GC‐MS for the five selected biomarkers. Paired t‐tests were utilized to statistically analyze the variations in VOCs in exhaled breath before and after exposure across the three groups.

### Fabrication and Characterization of Handheld Breath‐Analyzer

1. Comparison of the gas adsorption capability for various metallic oxides: The comparison of adsorption capability for the various metallic oxides (SnO_2_, WO_3_, Fe_2_O_3_, In_2_O_3_, NiO, ZnO, CeO_2_, TiO_2_, Cr_2_O_3_, Co_3_O_4_, Y_2_O_3_, and Mn_2_O_3_) to each studied gas is implemented by an analyzer of interface thermodynamic/kinetic parameters (ITKP, Xiamen High‐End MEMS Technology Co. Ltd., China).
2. Sensor fabrication: Details can be found in Supporting Information. In summary, the sensor was fabricated as a three‐electrode electrochemical sensor based on yttria‐stabilized zirconia (YSZ). It has a flat plate structure, with the sensing electrode (SE) and reference electrode (RE) all located on the same side of the plate, connecting to one end of printed silver wires. In this study, SnO_2_, WO_3_, Fe_2_O_3_, In_2_O_3_, NiO, and ZnO were used as the sensing electrode. In light of that the Mn‐based reference electrode (RE) is insensitive to all volatile organic compounds (VOCs), it allows for a simplified planar sensor design.^[^ ^27^ ^]^ Consequently, the Mn‐based RE was used in all of the fabricated sensors. The overall dimension of the sensor was 34 × 4 × 1 mm^3^. Denials can be found in Supporting Information.
3. Sensor characterization: Both SEs and Mn‐based RE of the sensor were simultaneously exposed to the base gas (diluted with air base) or the sample gas containing each of various VOCs to evaluate the gas sensing characteristics. Since the main research objective of this study is to develop a phenol sensor for the application of breath analysis, acetone, styrene, hexanal, ethanol, n‐butanol, ethylbenzene which are widely reported to be found in animal breath are selected as the interference gases. The base gas uses a volume ratio of 21% O_2_ mixed with N_2_ standard gas, maintaining a gas flow rate of 100 sccm (MFC: CS200 SCCM, Beijing Qixing Huachuang Electronics CO., Ltd). Three standard gases with a volume ratio of 50% O_2_ mixed with residual N_2_, phenol (or acetone, styrene, ethanol, n‐butanol, 2‐pentanone) mixed with residual N_2_ and 21% O_2_ mixed with residual N_2_ (Shanghai Weichuang Standard Gas Analysis Technology CO., Ltd.) were selected by using a mass flow controller (MFC, Beijing Qixing Huachuang Electronics CO., Ltd.) control the volume ratio of O_2_ to maintain at 21% and a total gas flow rate of 100sccm. The response signal (ΔV, ΔV = V_sample gas_ − V_base gas_) between SE and RE is recorded by using an electrometer (34970A, Agilent, USA). The operating temperature ranges from 400 to 500 °C. The carrier gas's background relative humidity (RH) was controlled by precisely blending dry air with air that was fully saturated with moisture (RH 100%). Hygrometer (4185 Traceable, USA) was used to monitor the ratio between the partial pressure of water vapor and the equilibrium vapor pressure of water in the mixture. The findings indicate that under steady environmental conditions within the range of 25–27 °C, the relative humidity test yields a result of ≈70%. All subsequent sensing test environments are conducted under the same temperature and humidity conditions.
4. Handheld breath‐analyzer construction and sensing breath markers

The handheld breath‐analyzer contains the following modules: sensor module (YSZ‐based phenol sensor), power module and data processing module (main cypher unit, MCU). Response difference is defined as: response signal = “Response signal measured in breath sample” – “Response signal measured in air”.

To continuously monitor the changes in breath markers following BoNT injection, a mouse was placed in a sealed glass container for breath sample collection. Oxygen was supplemented every 15 min using a glass syringe through a silicone plug, delivering 10 mL of pure oxygen (99.9%). A Teflon tube was connected directly from the glass container to a handheld breath analyzer, allowing for real‐time monitoring of breath marker variations.

### Fabrication and Characterization of Wearable Temperature Sensor

1. Fabrication of wearable temperature sensor based on GelMA film: In a brown bottle, added 20 mL of deionized water with 50 mg of the initiator LAP. Heated the mixture in a water bath at 40–50 °C, ensuring it was shielded from light, for 15 min. During this time, gently shake the bottle periodically until the initiator was completely dissolved, resulting in a clear 0.25% (w/v) initiator standard solution. Then, 1 g of GelMA was added to 10 mL of the prepared 0.25% (w/v) initiator standard solution. Placed the mixture in a water bath at 60–70 °C, again ensuring it was protected from light, and allowed it to heat for 20–30 min. The mixture was shaken intermittently until the GelMA was fully dissolved, producing a uniform 10% (w/v) GelMA solution. 100 mg of NaCl was introduced into the 10 mL of 10% (w/v) GelMA solution. The solution was heated in a water bath at 60–70 °C while stirring continuously, ensuring it remained shielded from light, for 5–10 min until the NaCl was completely dissolved, yielding a GelMA/NaCl solution. The GelMA/NaCl solution was spin‐coated on a glass substrate and further illuminated by UV for 30 seconds to form a GelMA/NaCl sensing film. To preserve the sensing performance of GelMA in air, glycerol was deliberately incorporated into the GelMA film to avoid the potential issue of dehydration.
2. Characterization of wearable temperature sensor: The sensor's electrical signals were recorded by electrometer (34970A, Agilent, USA). The environments for temperature‐sensing performance tests were created using a water bath (Herry Tech Co., Ltd., Shanghai) with an accuracy of ±0.1 °C. The current–voltage (*I*–*V*) curves were recorded on a CHI660E electrochemical analyzer (CH Instruments, Inc., Shanghai).

### Statistics

Experimental data on the Screening and Sensing characteristics towards volatile markers, detecting temperature of GelMA film and monitoring Abnormal Physiological Parameters were processed. At least three samples were tested for each data point. Mean values are shown in the plots and error bars correspond to standard deviations. T‐tests were used for statistical analysis of VOC markers, a p value of less than 0.05 (2‐sided significance testing) was considered statistically significant. Statistics were calculated and plotted using Excel and SPSS.

## Conflict of Interest

The authors declare no conflict of interest.

## Supporting information



Supporting Information

## Data Availability

Research data are not shared.
