# Supplementary material for: Wearable Temperature Sensor Enhanced Volatilomics Technique for Swift and Convenient Detection of Latrogenic Botulism
Source: Adv Sci (Weinh). 2024 Dec 16;12(6):2411738. doi: 10.1002/advs.202411738 (PMC11809335; doi:10.1002/advs.202411738)
Supplement: Supplementary file 1 — Supporting Information [file ADVS-12-2411738-s001.docx]

**Wearable Temperature Sensor Enhanced Volatilomics Technique for Swift and Convenient Detection of Latrogenic Botulism**

Xiaoyang Li^1,Ɨ^, Yufei Yan^2,Ɨ^, Chenyi Hu^1,Ɨ^, Jing Wang^1^, Jinlin Wang^1^, Hao Yang^1^, Daxiang Cui^2,3^, Wenwen Xin^1^, Shan Gao^1,*^, Han Jin^3,4,5,*^

^1^ State Key Laboratory of Pathogen and Biosecurity, Academy of Military Medical Sciences, Beijing 100071, China

^2^ Institute of Micro-Nano Science and Technology & National Key Laboratory of Advanced Micro and Nano Manufacture Technology, School of Electronic Information and Electrical Engineering, Shanghai Jiao Tong University, Shanghai 200240, P. R. China

^3^ Medical School, Henan University, Kaifeng 475004, Henan Province, P. R. China.

^4^ National Engineering Research Center for Nanotechnology, Shanghai 200241, P. R. China.

^5^ Wuzhen Laboratory, Tongxiang 314500, Zhejiang Province, P. R. China.

E-mail addresses: gaoshan845@163.com(Shan Gao)

jinhan10@sjtu.edu.cn (Han Jin)

^Ɨ^ These authors contribute equally.

**Supplementary Methods:**

Details of sensor fabrication and sample gas preparation

1. Sensor preparation process

YSZ-based electrochemical phenol sensor fabrication processes are as follows:

1) Binder preparation: mix turpentine alcohol and ethyl cellulose in a ratio of 94:6 by mass to prepare a turpentine alcohol slurry;

2) Preparation of reference electrode: grind a mixture of turpentine alcohol slurry and manganese dioxide powder in a mass ratio of 1.2:1 using an agate mortar to obtain a reference electrode slurry. Coat the reference electrode slurry onto the reference electrode lead based on yttria-stabilized zirconia;

3) Forming reference electrodes: The sensor are first dried at 130°C for 4 hours and then placed in a muffle furnace for sintering. The sintering temperature profile was as follows: ramp up from room temperature to 1400°C at a rate of 3°C/min, hold at 1400°C for 2 hours, and then naturally cool to room temperature after the sintering process;

4) Preparation of the sensing electrode: grind a mixture of turpentine alcohol slurry and various oxides powder in a mass ratio of 1:1 using an agate mortar to obtain a sensing electrode slurry. Coat the sensing electrode slurry onto the lead of the sensing electrode;

5) Forming sensing electrode: the sensor are first dried at 130°C for 4 hours and placed in a muffle furnace for sintering. The sintering temperature profile is as follows: ramp up from room temperature to 1050°C at a rate of 3°C/min, hold at 1050°C for 2 hours, and then naturally cool to room temperature after the sintering process.

(2) Details of sample gas preparation

The testing system mainly consists of the sensor, quartz testing chamber, digital multimeter, power supply, gas cylinders, and a gas distribution system. The gas distribution system has nine interfaces, which can be connected to nine gas cylinders (replaceable) through pressure-reducing valves. The gas switches and flow direction were controlled by two-way/three-way valves in the pipeline. The gas flow rate was controlled by seven mass flow controllers (MFC) arranged in the pipeline. The real-time flow was adjusted and monitored by the Muti-Digital MFC software on the computer. The digital multimeter was connected to the computer through an I/O interface transmission line, and the Agilent signal acquisition software was used to collect and display the response curve of the sensor in real time. The entire system can dynamically configure gases of different concentrations and compositions, exposing the sensor to the atmosphere of the test gas to achieve real-time collection of response signals.

To align with real-world application scenarios, the sensor was exposed to a simulated air environment, and the response signal in this environment was considered as the baseline state for the sensor. The main principle of sensor testing relies on the redox reactions at the interface, directly correlated with the concentration of oxygen. In air, the primary factor affecting the sensor response signal is the concentration of oxygen. Therefore, the simulated air contains 21% oxygen, with the rest being nitrogen. Additionally, when diluting samples of different concentrations or creating gas mixtures, it is essential to ensure that the final oxygen concentration in the gas is 21%. For dilution, three gases are used: the original test gas, 50% oxygen, and air. The total gas flow rate was maintained at 100 sccm, and the proportions of different gases in the mixture were controlled by the gas flow rates. This was achieved through mass flow controllers to adjust the gas concentration, with the calculation formula as follows:

$$\frac{C}{C_{0}}=\frac{x}{100}$$

$$0.21y+0.5z=21$$

$$x+y+z=100$$

Where *C_0_* is the original concentration of the test gas, *C* is the target concentration of the test gas; x, y, and z represent the actual gas flow rates of the test gas, air, and oxygen, respectively. Based on the above principal formula, the numerical values of the flow rates for the three required gases during gas dilution can be obtained as follows:

$$x=\frac{100C}{C_{0}}$$

$$y=100-\frac{5000C}{29C_{0}}$$

$$z=\frac{2100C}{29C_{0}}$$

**Supplementary Figures:**

1. Representative chromatograms of mice following a) ricin (RT); b) tetrodotoxin (TTX) injection；c) suffering anoixa and healthy mice; 1,2: acetone; 3,4: ethanol; 5,6: 2-pentanone; 7,8:n-butanol; 9,10: styrene. (Figure S1)
2. Sensing performance of the sensors fabricated at different calcination temperature (Figure S2)
3. Sensing performance of the sensors at different operational temperature (Figure S3)
4. Response patterns of the sensors using CeO_2_, TiO_2_, Cr_2_O_3_, Co_3_O_4_,Y_2_O_3_ and Mn_2_O_3_-SEs (vs. Mn-based RE) toward the targeted volatile markers at 1ppm (Figure S4)
5. 90% response/recovery time of various sensors using SnO_2_, WO_3_, Fe_2_O_3_, In_2_O_3_, NiO and ZnO (vs. Mn-based RE) (Figure S5)
6. Response stability of the developed sensors within 1 month (Figure S6)
7. Variation of the response signal for the developed sensors at different humidity (Figure S7)
8. Comparison of the bio-compatibility for ecoflex or GelMA film (Figure S8)
9. Photographic images of a) handheld breath analyzer and b) wearable temperature sensor; comparison of data deviation for the developed c) breath analyzer and d) wearable temperature sensor (Figure S9)
10. Schematic diagram of the circuit board for the handheld breath-analyzer ( Figure S10)
11. Schematic diagram of the circuit board for the wearable temperature sensor (Figure S11)
12. Illustration of the PCA patterns that obtained from a) body temperature signal; b) breath signal; c) combined signal (Figure S12)

**Supplementary table:**

1. Summary of volatile markers under BoNT, ricin, tetrodotoxin poisoning and hypoxia (Table S1)
2. Presence of target breath markers identified under various physical conditions (Table S2)

**
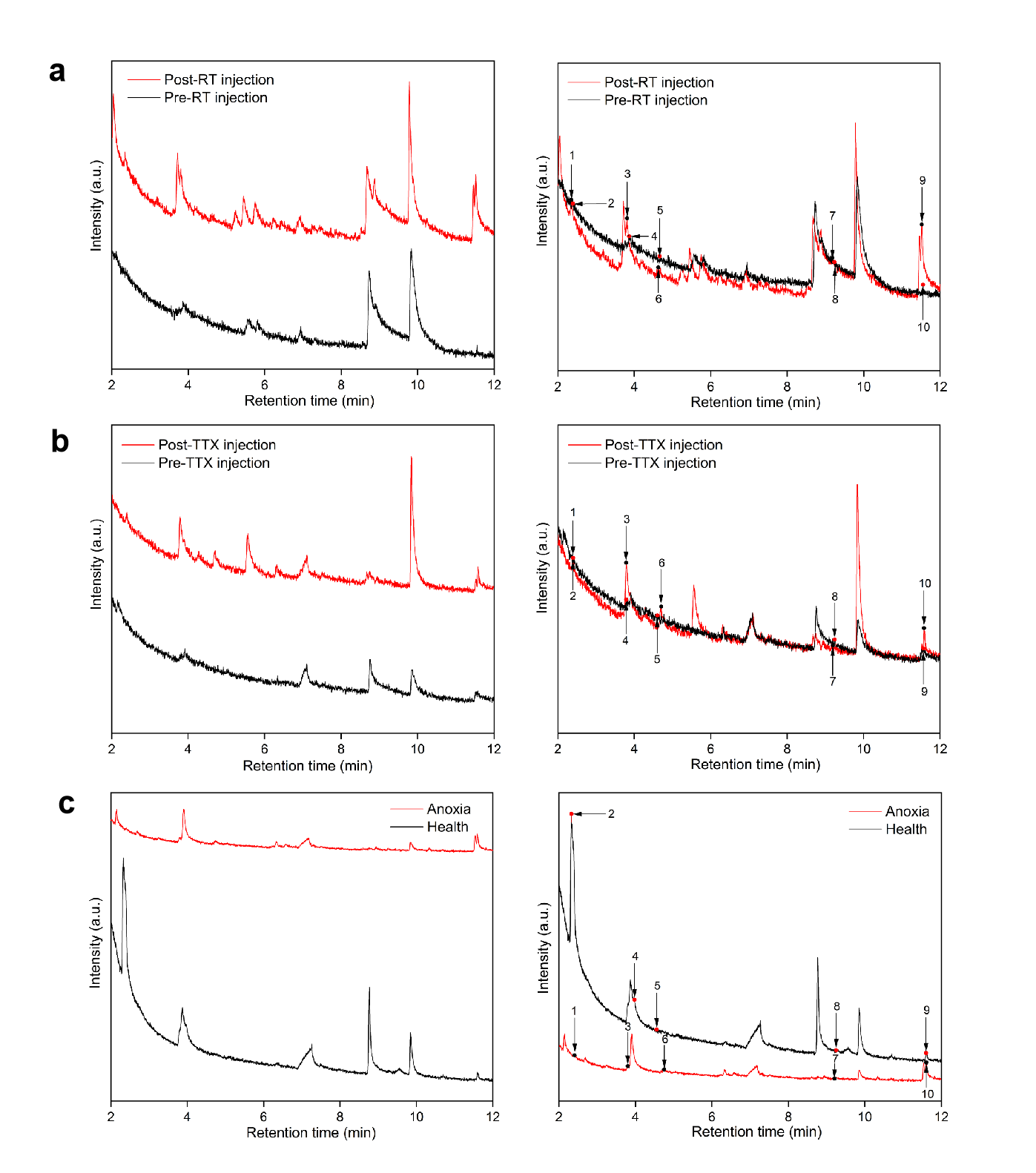
Figure S1.** Representative chromatograms of mice following a) ricin (RT); b) tetrodotoxin (TTX) injection；c) suffering anoixa and healthy mice; 1,2: acetone; 3,4: ethanol; 5,6: 2-pentanone; 7,8:1-butanol; 9,10: styrene

**Figure S2.**
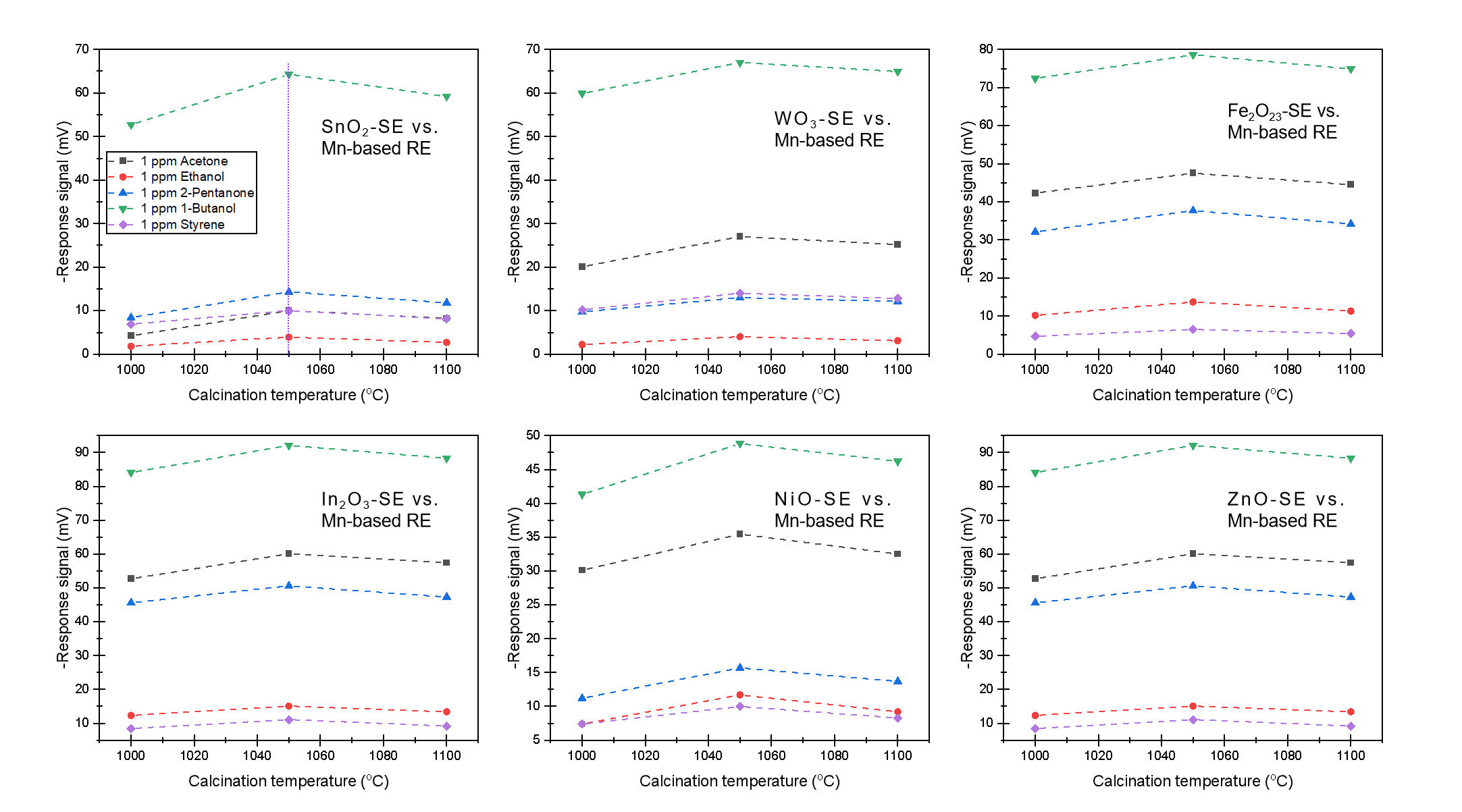
 Sensing performance of the sensors fabricated at different calcination temperature


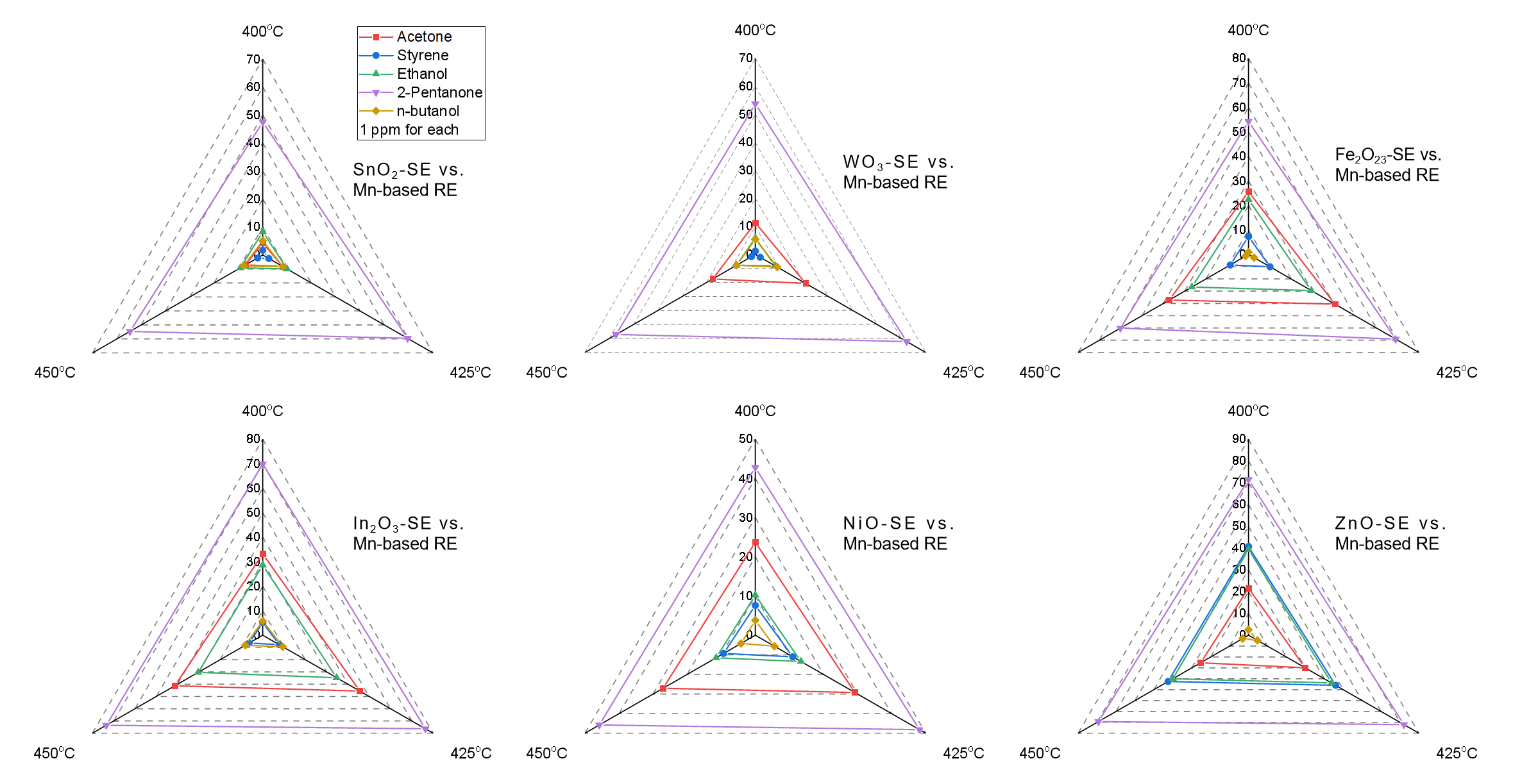
**Figure S3.** Sensing performance of the sensors at different operational temperature

**
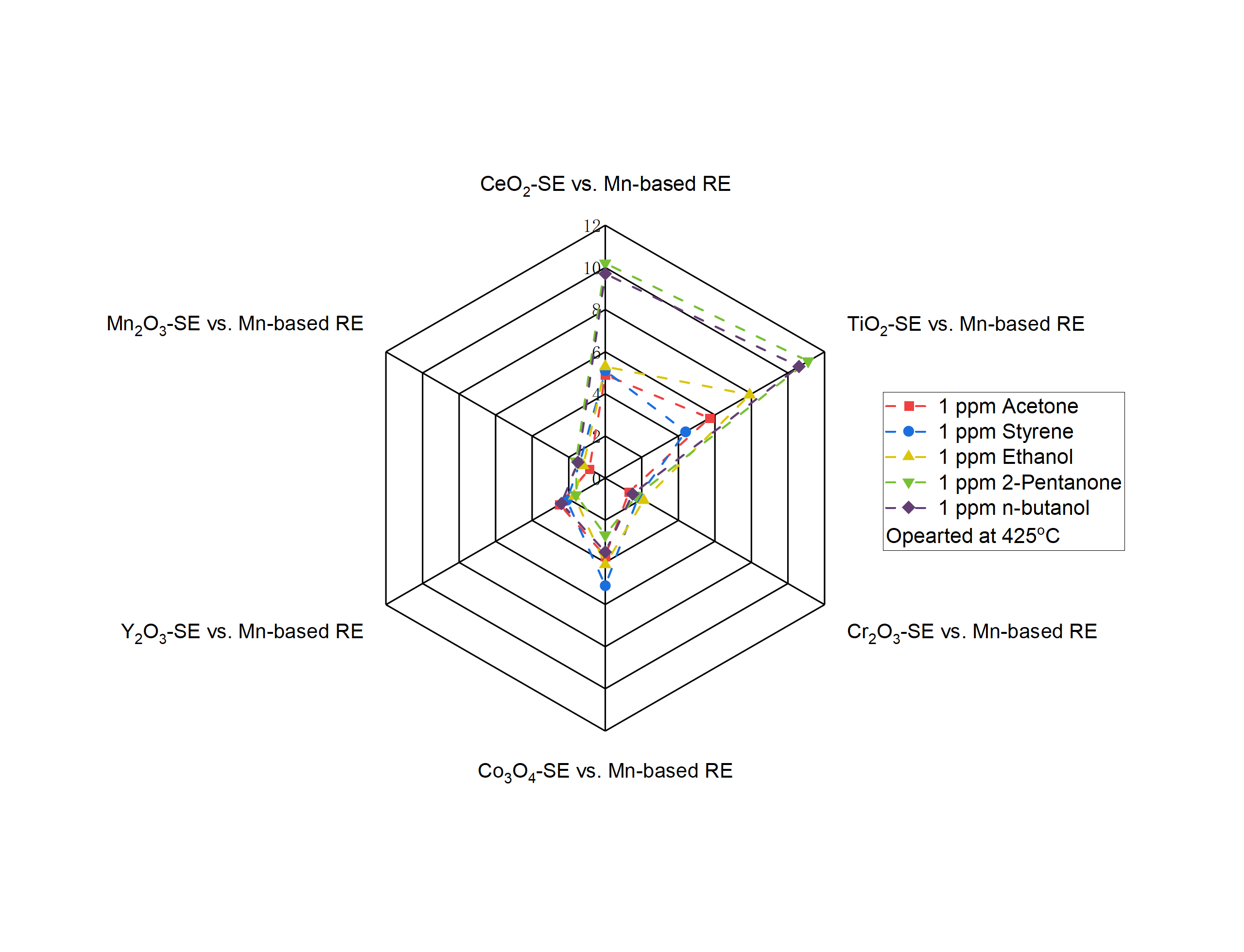
Figure S4.** Response patterns of the sensors using CeO_2_, TiO_2_, Cr_2_O_3_, Co_3_O_4_,Y_2_O_3_ and Mn_2_O_3_-SEs (vs. Mn-based RE) toward the targeted volatile markers at 1ppm


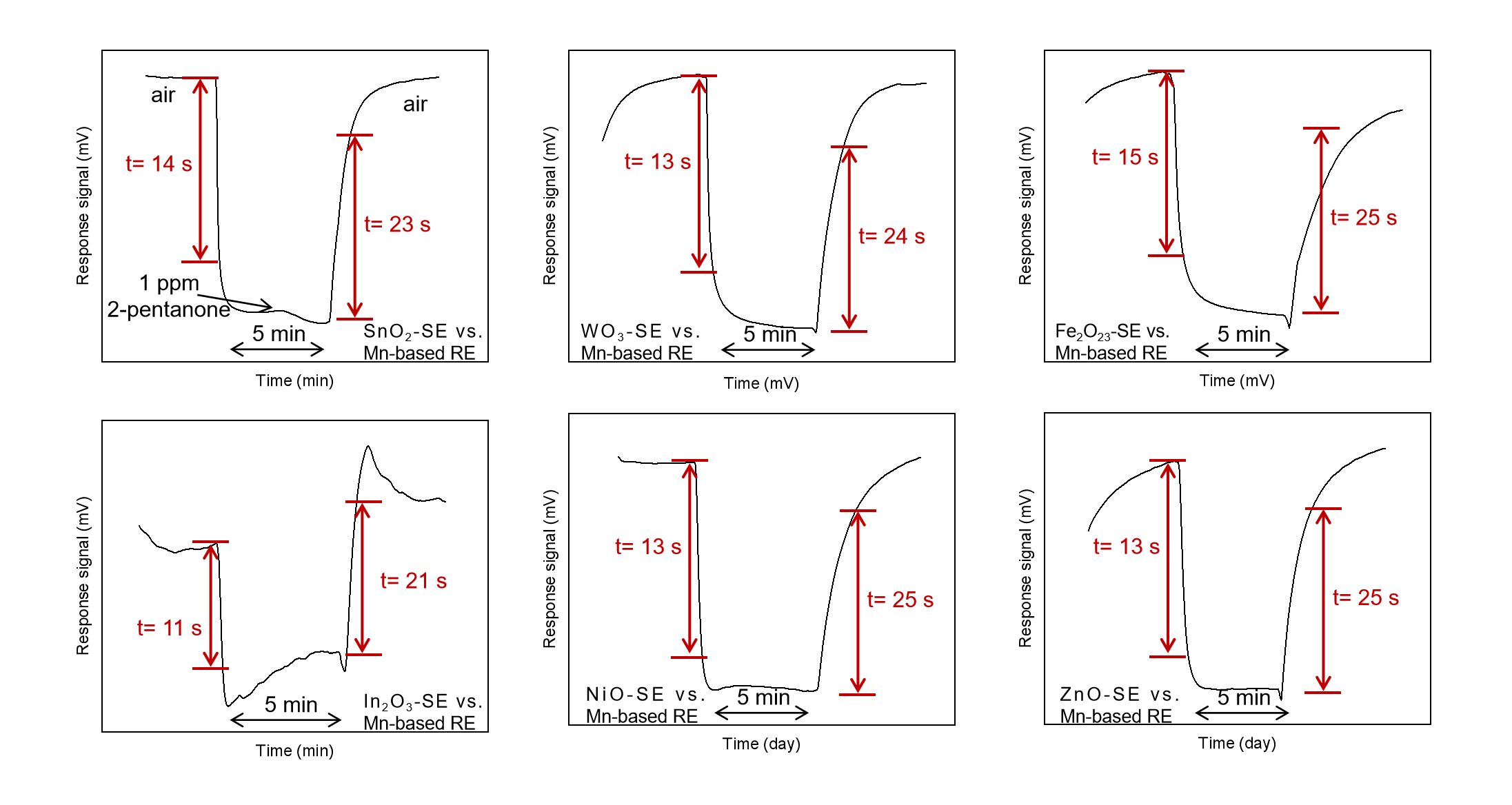
**Figure S5.** 90% response/recovery time of various sensors using SnO_2_, WO_3_, Fe_2_O_3_, In_2_O_3_, NiO and ZnO (vs. Mn-based RE)

**Figure S6.** Response stability of the developed sensors within 1 month

**Figure S7.** Variation of the response signal for the developed sensors at different humidity


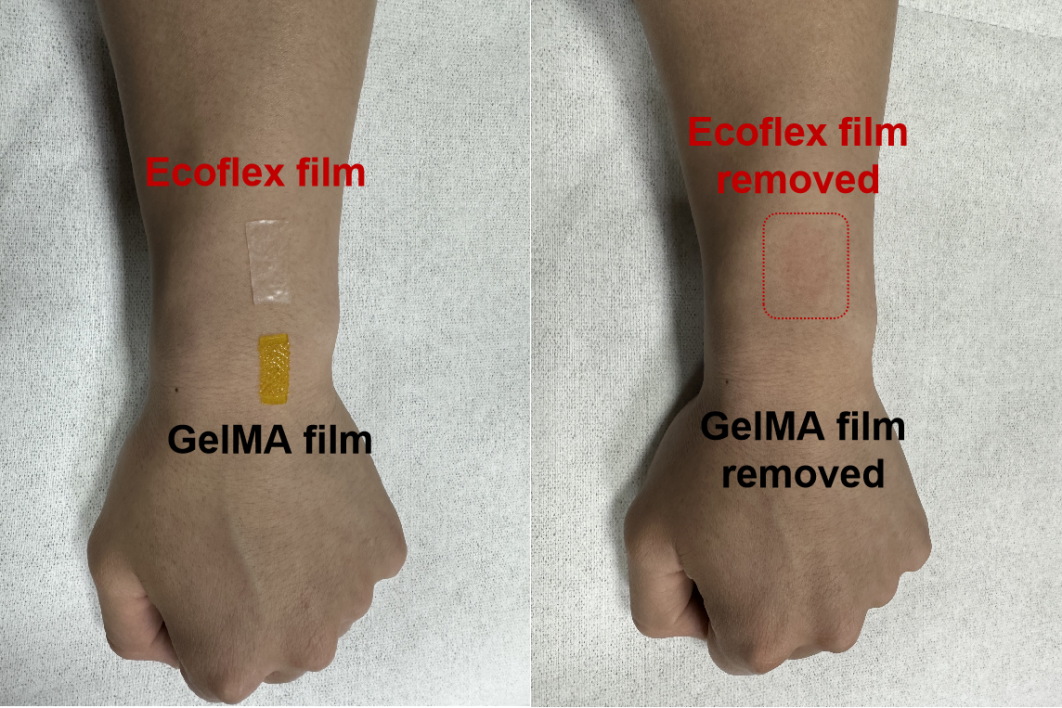
**Figure S8.** Comparison of the bio-compatibility for ecoflex or GelMA film


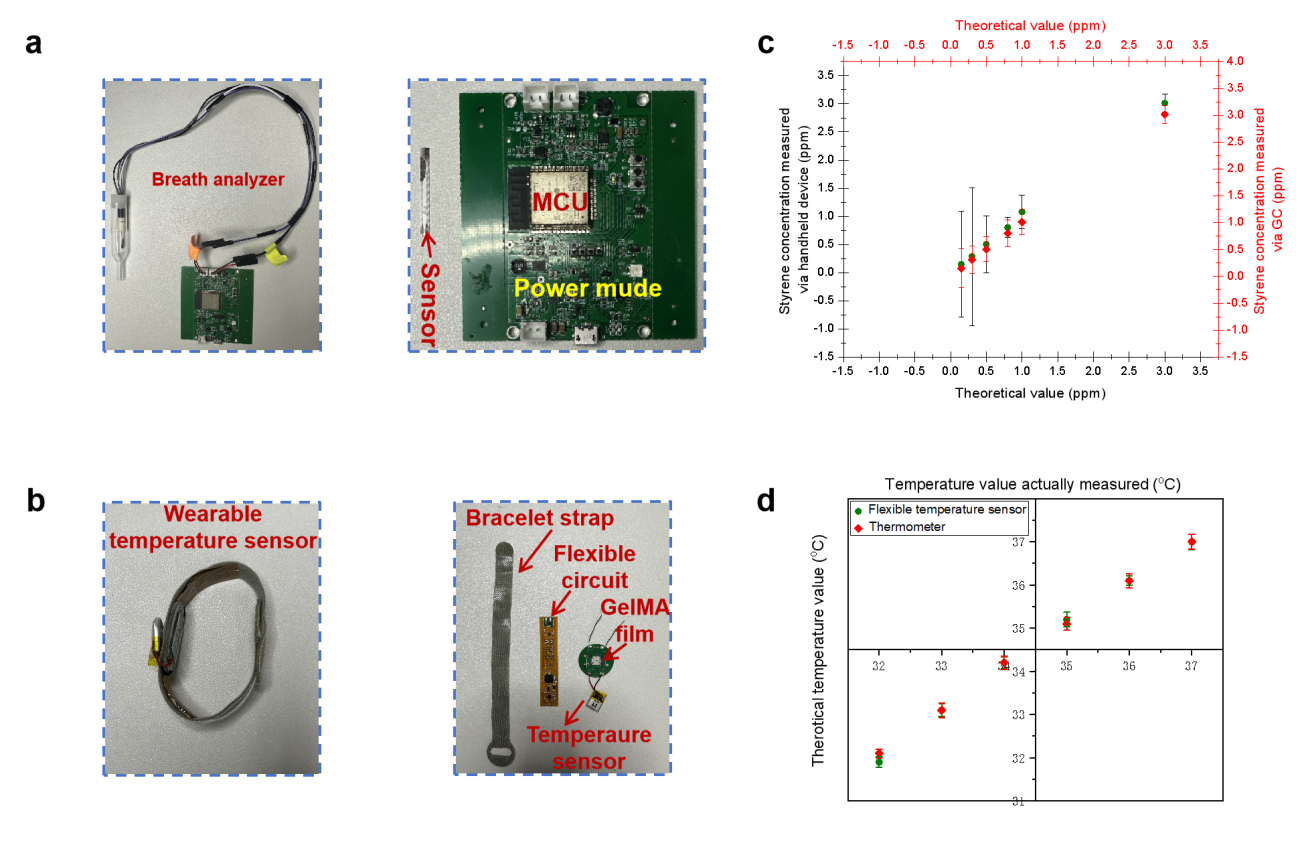
**Figure S9.** Photographic images of a) handheld breath analyzer and b) wearable temperature sensor; comparison of data deviation for the developed c) breath analyzer and d) wearable temperature sensor


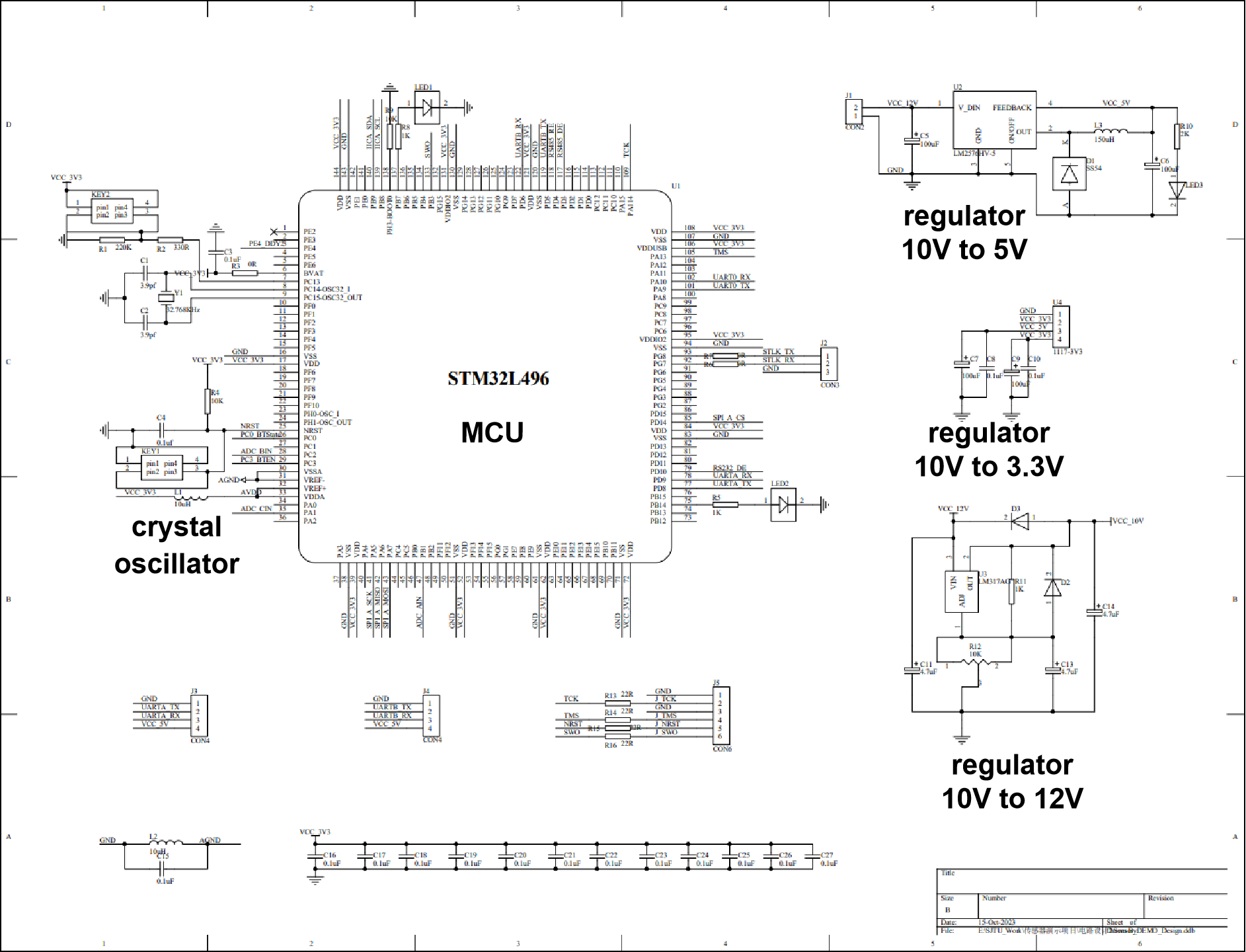
**Figure S10.** Schematic diagram of the circuit board for the handheld breath-analyzer


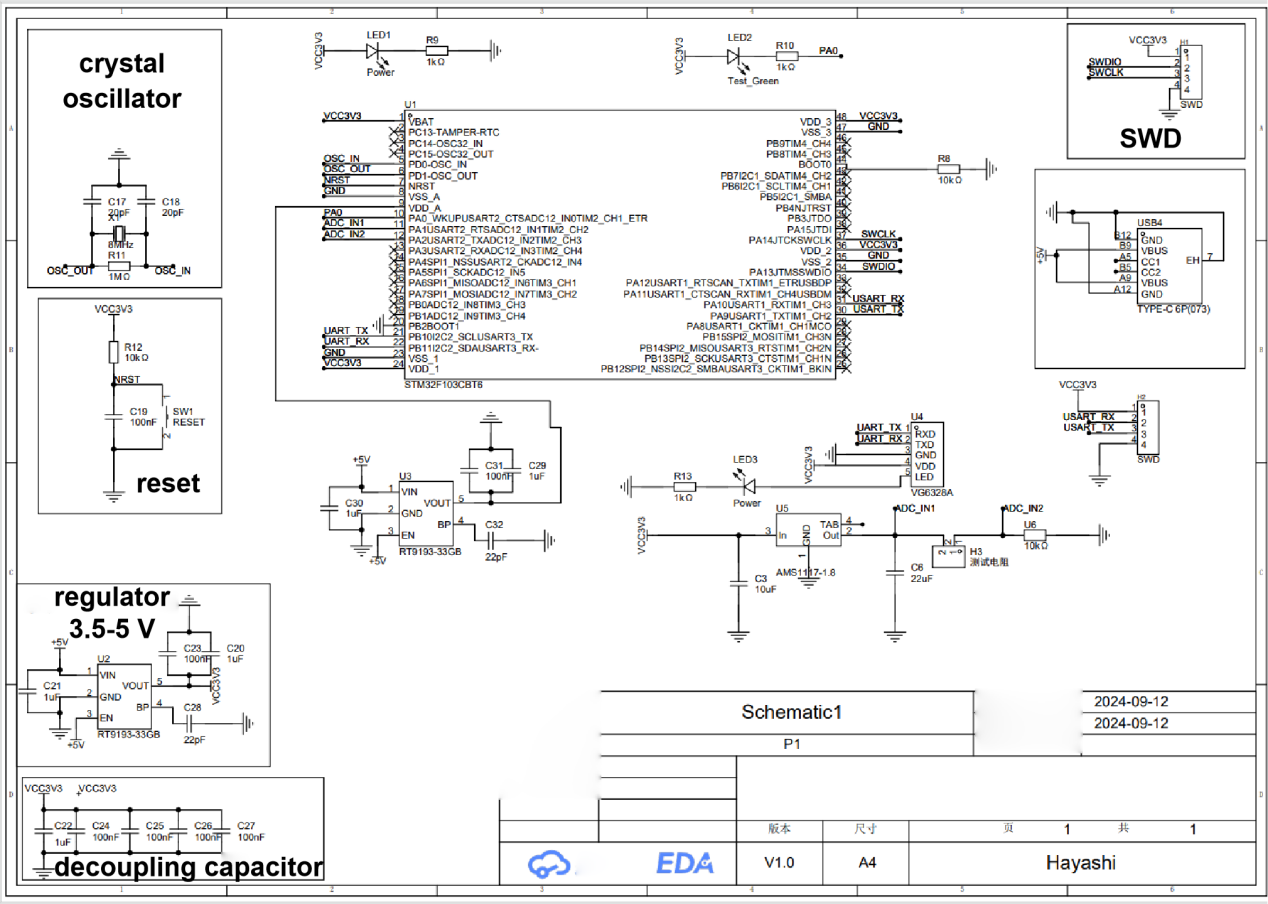
**Figure S11.** Schematic diagram of the circuit board for the wearable temperature sensor


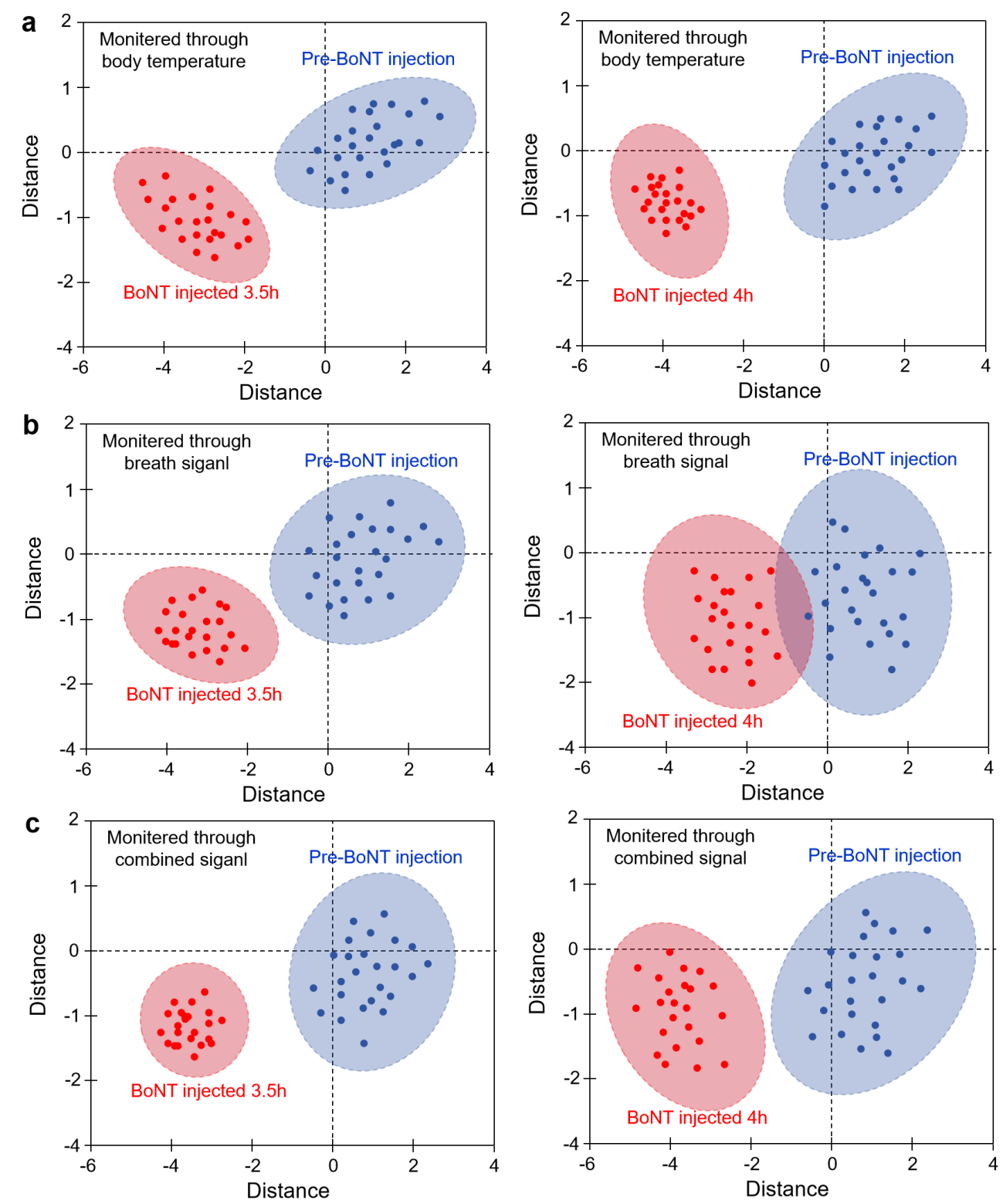


**Figure S12.** Illustration of the PCA patterns that obtained from a) body temperature signal; b) breath signal; c) combined signal

**Table S1:** Summary of volatile markers under BoNT, ricin, tetrodotoxin poisoning and hypoxia

| **Method** | **Volatile markers** | **Peak intensity mean value** | **Standard deviation** | **Standard error of Mean** | **95% Confidence Interval** | | **Sig.** |
| --- | --- | --- | --- | --- | --- | --- | --- |
|  |  |  |  |  | **lower-bound** | **upper-bound** |  |
| Injecting botulinum toxin  (BoNT) | acetone | -14551.4 | 8967.73895 | 4010.49478 | -25686.31859 | -3416.48141 | 0.022 |
|  | ethanol | -28946.2 | 20868.20406 | 9332.54457 | -54857.49769 | -3034.90231 | 0.036 |
|  | 2-Pentanone | -9183 | 7079.15235 | 3165.89318 | -17972.92861 | -393.07139 | 0.044 |
|  | 1-Butanol | -8383.4 | 3148.45649 | 1408.03255 | -12292.72508 | -4474.07492 | 0.004 |
|  | styrene | -16918.8 | 12468.19745 | 5575.94741 | -32400.1119 | -1437.4881 | 0.039 |
| Hypoxia | acetone | -113505 | 260203.0408 | 116366.3374 | -436589.748 | 209579.748 | 0.385 |
|  | ethanol | -854.8 | 15792.38233 | 7062.56808 | -20463.63259 | 18754.03259 | 0.91 |
|  | 2-Pentanone | -1546 | 1286.56655 | 575.37005 | -3143.48337 | 51.48337 | 0.055 |
|  | 1-Butanol | -957 | 3300.59472 | 1476.07083 | -5055.22964 | 3141.22964 | 0.552 |
|  | styrene | -37657.4 | 72204.3267 | 32290.75655 | -127310.913 | 51996.11298 | 0.308 |
| Injection Ricin  (RT) | acetone | -858.4 | 2994.14801 | 1339.0237 | -4576.12579 | 2859.32579 | 0.556 |
|  | ethanol | -868.2 | 3234.13933 | 1446.35108 | -4883.91437 | 3147.51437 | 0.581 |
|  | 2-Pentanone | -269.8 | 631.13366 | 282.25155 | -1053.45595 | 513.85595 | 0.393 |
|  | 1-Butanol | -589.6 | 849.23689 | 379.79028 | -1644.06688 | 464.86688 | 0.196 |
|  | styrene | -4635.4 | 4789.73635 | 2142.03521 | -10582.64319 | 1311.84319 | 0.096 |
| Injecting Tetrodotoxin  (TTX) | acetone | -2832.2 | 2372.45805 | 1060.99549 | -5777.99575 | 113.59575 | 0.056 |
|  | ethanol | 618.2 | 1881.34545 | 841.36326 | -1717.79891 | 2954.19891 | 0.503 |
|  | 2-Pentanone | -2311 | 1002.6475 | 448.39759 | -3555.9513 | -1066.0487 | 0.007 |
|  | 1-Butanol | -1245.8 | 1603.08007 | 716.9192 | -3236.2868 | 744.6868 | 0.157 |
|  | styrene | -9835.8 | 8799.77399 | 3935.37856 | -20762.16255 | 1090.56255 | 0.067 |

*Utilizing the paired t-test method and analyzed with SPSS software, a p-value ≤ 0.05 signifies that the observed difference is statistically significant.

**Table S2:** Presence of target breath markers identified under various physical conditions

| Physical state  Volatile markers | BoNT poisoning | Hypoxia | RT poisoning | TTX poisoning |
| --- | --- | --- | --- | --- |
| Acetone | + | － | － | － |
| Ethanol | + | － | － | － |
| 2-pentanone | + | － | － | + |
| n-butanol | + | － | － | － |
| Styrene | + | － | + | － |

(+：t≤0.05；-：t＞0.05)
